# Design and development of a phantom for tomosynthesis with potential for automated analysis via the cloud

**DOI:** 10.1002/acm2.12297

**Published:** 2018-03-06

**Authors:** David Goodenough, Josh Levy, Hildur Olafsdottir, Ingvi Olafsson

**Affiliations:** ^1^ Department of Radiology The George Washington University NW Washington DC USA; ^2^ The Institute For Radiological Image Sciences Myersville MD USA; ^3^ The Phantom Laboratory Salem NY USA; ^4^ Image Owl Salem NY USA; ^5^ Raförninn Reykjavík Iceland

**Keywords:** Mammography, QA Phantoms, Tomosynthesis

## Abstract

This paper describes Development of a Phantom for Tomosynthesis with Potential for Automated Analysis via the Cloud. Several studies are underway to investigate the effectiveness of Tomosynthesis Mammographic Image Screening, including the large TMIST project as funded by the National Cancer Institute https://www.cancer.gov/about-cancer/treatment/clinical-trials/nci-supported/tmist. The development of the phantom described in this paper follows initiatives from the FDA, the AAPM TG245 task group, and European Reference Organization (EUREF) for Quality Assured Breast Screening and Diagnostic Services Committee report noting, that no formal endorsement nor recommendation for use has been sought, or granted by any of these groups. This paper reports on the possibility of using this newly developed Tomosynthesis Phantom for Quality Assurance, field testing of image performance, including remote monitoring of DBT system performance, e.g., via transmission over the cloud. The phantom includes tests for: phantom positioning and alignment (important for remote analysis), scan geometry (x and y), chest wall offset, scan slice width and Slice Sensitivity Profile (SSP(z)) slice geometry (slice width), scan slice incrementation (z), z axis geometry bead, low contrast detectability using low contrast spheres, spatial resolution via Point Spread Function (PSF), Image uniformity, Signal to Noise Ratio (SNR), and Contrast to Noise Ratio (CNR) via readings over an Aluminum square. The phantom is designed for use with automated analysis via transmission of images over the cloud and the analysis package includes test of positioning accuracy (roll, pitch, and yaw). Data are shown from several commercial Tomosynthesis Scanners including Fuji, GE, Hologic, IMS‐Giotti, and Siemens; however, the focus of this paper is on phantom design, and not in general aimed at direct commercial comparisons, and wherever possible the identity of the data is anonymized. Results of automated analysis of the phantom are shown, and it is demonstrated that reliable analysis of such a phantom can be achieved remotely, including transmission of data through the cloud.

## 1 INTRODUCTION

The recent development of Tomosynthesis,^1^, ^2^ and in particular the commercialization of Digital Breast Tomography (DBT) has led to interest in developing Phantoms for assessment of image quality as well as Quality Assurance (QA). Several studies are underway to investigate the effectiveness of Tomosynthesis Mammographic Image Screening, including the large TMIST project as funded by the National Cancer Institute https://www.cancer.gov/about-cancer/treatment/clinical-trials/nci-supported/tmist.

This work describes a new phantom,^3^ the Tomophan^®^ (The Phantom Laboratory, Salem, NY, USA), which was introduced commercially at the end of 2016. This work was stimulated by initiatives from the FDA,^4^ the AAPM TG245 task group, and “Protocol for the Quality Control of the Physical and Technical Aspects of Digital Breast Tomosynthesis Systems”, 2013, European Reference Organization (EUREF) for Quality Assured Breast Screening and Diagnostic Services, Committee Report although no formal endorsement nor recommendation for use has been sought, or granted by either group. This paper reports on the possibility of using this newly developed Tomosynthesis Phantom for field testing, QA, and research. The paper also reports on some of the results of remote monitoring of DBT sites, e.g., via transmission over the Cloud utilizing a commercial service (Image Owl, Greenwich, NY, USA and Reykjavik, Iceland). This paper discusses much of the scientific foundation for the phantom, as opposed to the user manual which provides instructions for the use of the phantom.

Differences between the Tomophan and EUREF design, and other phantoms as described by the FDA are pointed out. In particular, the study of the slice sensitivity profile by use of the angled bead ramps in the Tomophan vs. the stepped plates of the EUREF phantom, and the use of the small beads as Point Sources (for the Point Spread Function – PSF) as a method to determine the Modulation Transfer Function (MTF),^5^ as opposed to use of line sources in other (FDA poster) design.

## 2 MATERIALS AND METHODS

**Figure 1:**
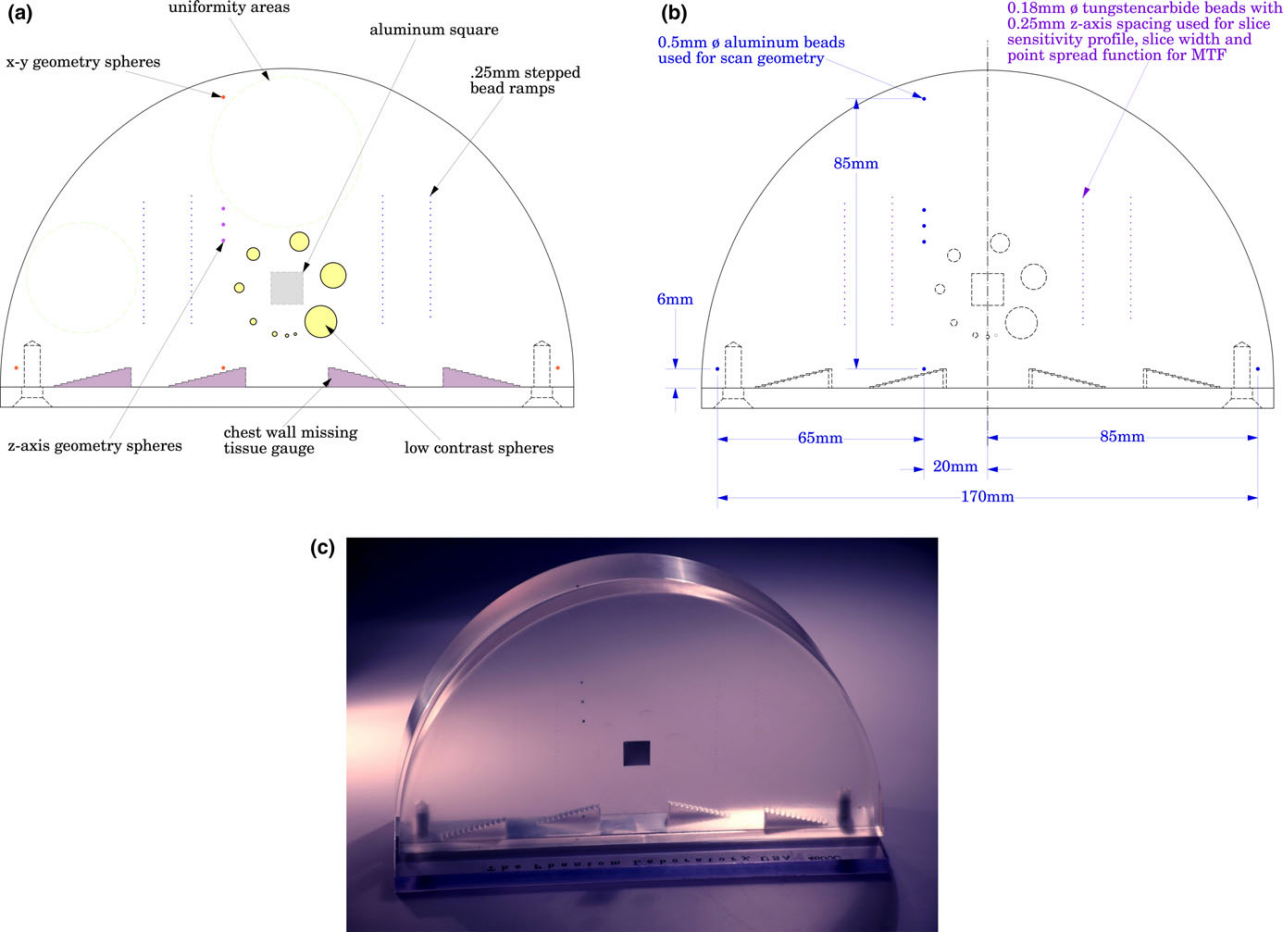
Schematic overview of the Tomophan. (a) Top view of phantom, showing component test objects. (b) Spacing and dimensional data for the phantom. (c) Photograph of Phantom.

A newly developed Tomosynthesis QA Phantom (Tomophan^®^, Salem, NY, USA) has been used for testing DBT systems. This Phantom (Fig. 1) is designed to be responsive to a number of scientific and regulatory groups, including those mentioned in the introduction and other international groups.^6^ The phantom is also designed to allow remote analysis via web or cloud. The phantom includes tests for: phantom positioning and alignment (important for remote analysis), scan geometry (x and y), chest wall offset, Slice Sensitivity Profile (SSP(z)), slice geometry (slice width), scan slice incrementation (z), z axis geometry bead, low contrast detectability using low contrast spheres, spatial resolution via Point Spread Function (PSF) and the corresponding Fourier Transform yielding the MTF, Image uniformity, Signal to Noise Ratio (SNR), and Contrast to Noise Ratio (CNR) via readings over the Aluminum square.

**Figure 2:**
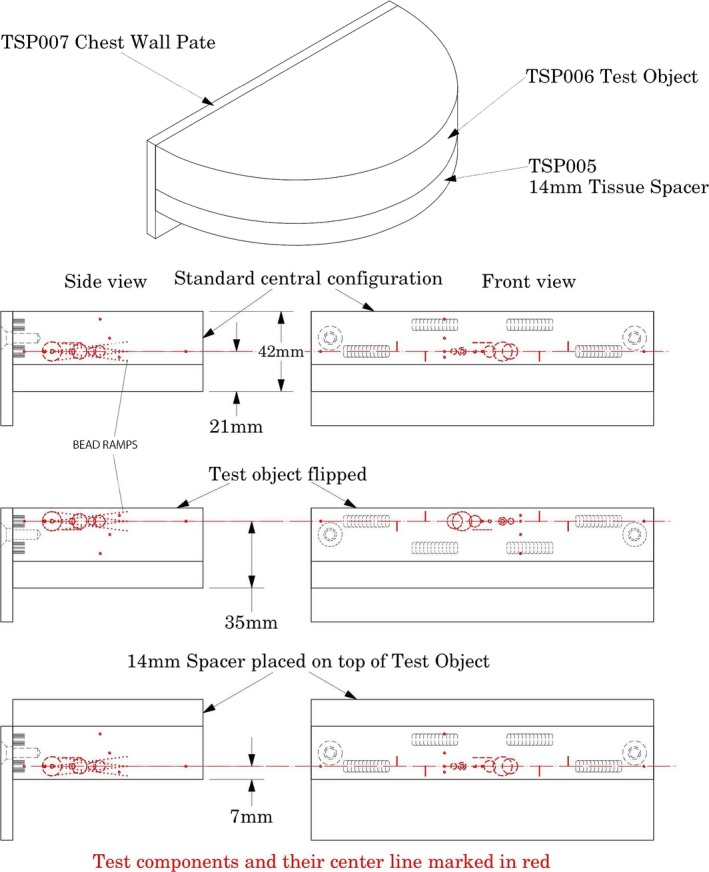
Various configurations of the test objects and the 14 mm tissue spacer shown in front and side views.

The Tomophan (TSP) phantom is comprised of three components: the TSP006 Test Object (see Fig. 2); TSP005, a 14 mm Tissue Spacer; and TSP007, Chest Wall Plate. In the standard configuration, the test components are in the central plane of the phantom. These components can be configured in different positions to allow testing slices in the upper central and lower region of the assembly's 42 mm thickness.

**Figure 3:**
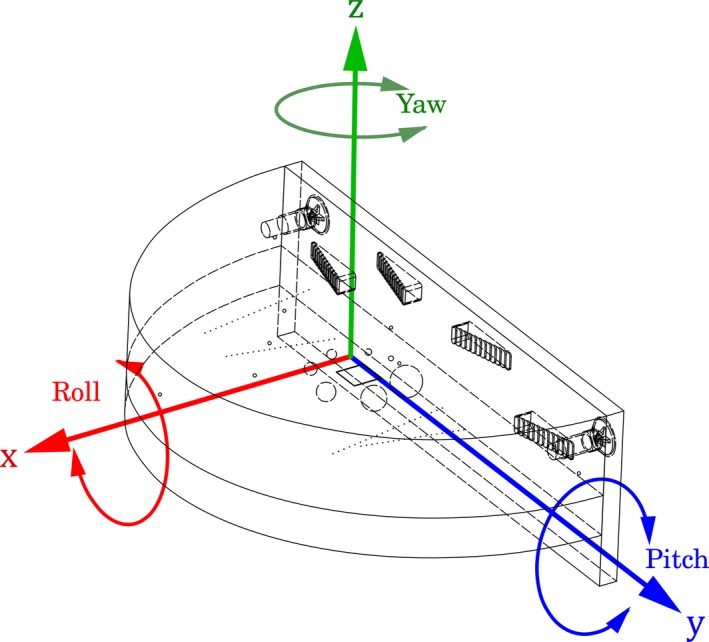
Illustration of the three positional tests (yaw, role, and pitch) that are determined in the automated test software.

Additionally, the phantom has components so that roll (rotation), pitch, and yaw can be calculated as a method of monitoring or controlling phantom positioning effects (Fig. 3).

The phantom has been used with several commercially available DBT systems (GE, Hologic, Siemens, Fuji, IMS) to study several aspects of image quality performance. The phantom was scanned at the appropriate site and then the image data was uploaded to the Image Owl cloud platform for automated image processing (tomo.imageowl.com).

Results were returned within seconds to the originating site for review by the local medical physics and clinical staff.

### 2.A Design of specific test and initial results

In the following section, results are shown for monitoring several of the parameters as listed in Methods and Materials. Data are shown for several commercial Tomosynthesis systems. In future publications, system reproducibility and performance over time, and longitudinal monitoring will also be reported. Examples can also be found on the Image Owl website (tomo.imageowl.com).

## 3 RESULTS AND DISCUSSION

### 3.A Chest wall offset (missing tissue)

**Figure 4:**
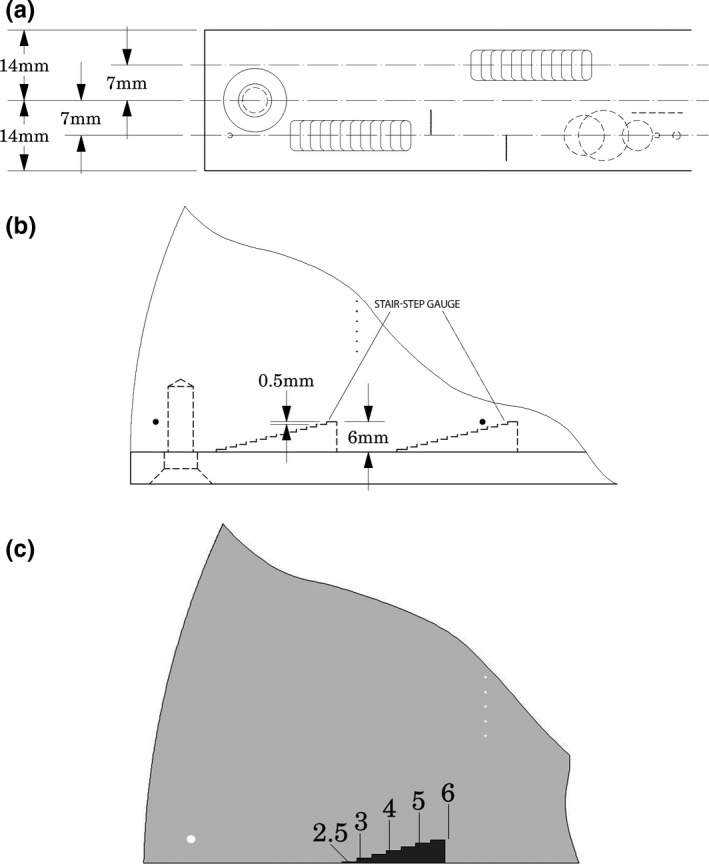
Chest Wall offset (missing tissue). (a) Side view (b) Dimensioned view of stair‐step gauges. (c) Expanded view of an actual scan showing slightly less than 2.5 mm of chest wall loss.

A number of approaches can be used to study the offset, including an independent step wedge. The relevant section of this phantom for determination of chest wall offset (missing tissue) is shown in Fig. 4(a), with an expanded view illustrating how the stair‐step gauges are used to measure missing tissue (distance to chest wall).^7^ The four gauges have 12 steps in 0.5 mm increments rising into the phantom from the chest wall. In the case of Fig. 4(b) an illustration is shown for two locations along the wall at a given reconstructed slice thickness. Likewise Fig. 4(c) illustrates how a gauge in expanded view, shows how many steps are seen. The image in Fig. 4(c), thus represents slightly less 2.5 mm of chest wall being lost in the image.

**Figure 5:**
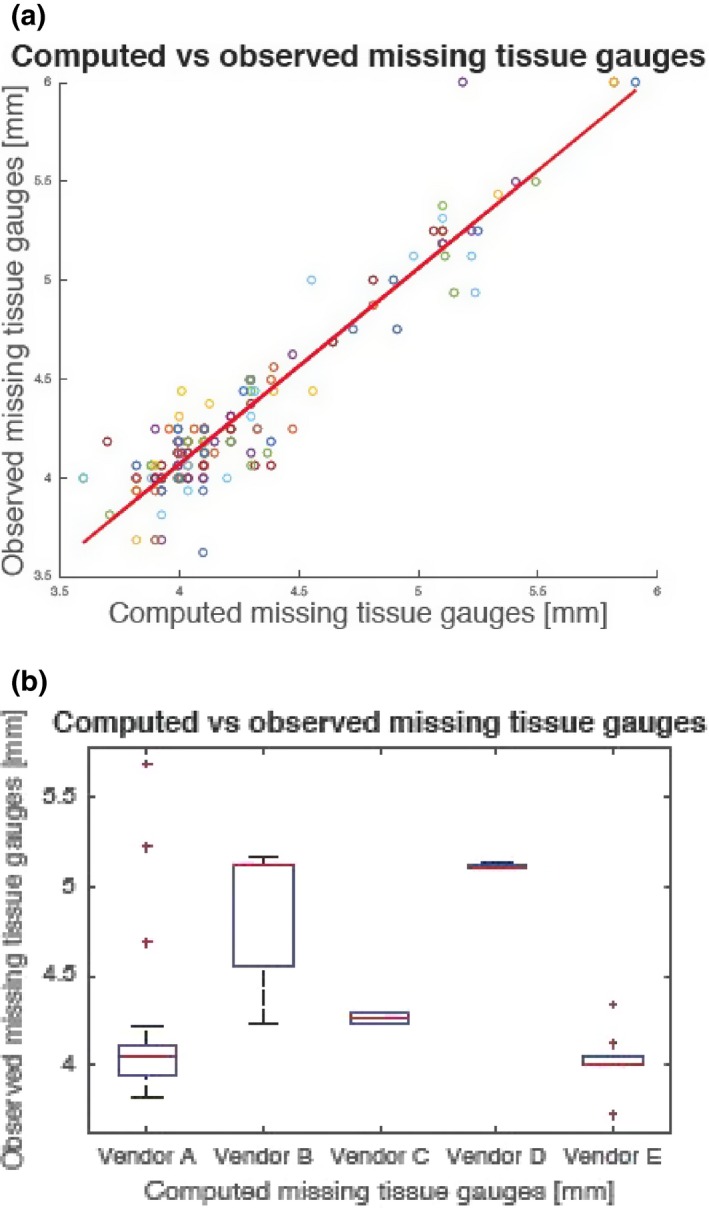
Computed vs. observed amounts of missing tissue using the chest wall offset gauges. (a) Data combined from five vendors (a through e). (b) Data from each vendor (a through e).

The phantom was imaged in Digital Breast Tomosynthesis (DBT) systems from various vendors (A–E) resulting in 46 cases used for testing. The results in Fig. 5(a) show that on average 1.9 mm of 6 mm of the gauges are visible, resulting in about 4.1 mm of missing tissue. On each box, the central mark is the median, the edges of the box are the 25th and 75th percentiles, the whiskers extend to the most extreme data points the algorithm considers not to be outliers, and the outliers are plotted individually (red ‘+’ marker). A small focus group of several people (engineers, physicists, and technologists) was asked to count the number of visible steps for each case which resulted in a good agreement between observer counts of steps and computed data from Image Owl where steps are located and measured. No formal assessment (statistical test) of level of agreement was made because this was just a preliminary finding.

### 3.B Slice sensitivity profile – slice thickness, and slice position

**Figure 6:**
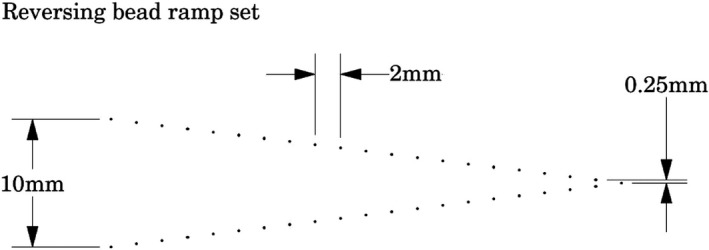
Side view of one of the two reversing bead ramps. Note that the upper and lower sections of the folded bead ramps are offset laterally at the junction point.

One of the items of interest in DBT is the nature of the slice thickness position and sensitivity profile. Aspects of the effects of these tests, are revealed by the Artifact Spread Function (ASF).^4^ This topic is discussed in the subsequent section. In the design of the tests in this phantom use is made of the well‐established technique of trigonometric projection of z‐axis depth onto the x‐y plane.^8^ The DBT phantom includes two sets of two (folded) angled bead ramps (left and right of center) Fig. 2. Each set has two ramps (laterally offset) with the top bead of the lower ramp centered .25 mm below the bottom bead in the upper ramp (see illustrations below). The diameter of each bead is 0.18 mm, and beads are spaced vertically at 0.25 mm. The size of the bead is small enough that it essentially constitutes a Point Source for current DBT resolution (reconstructed slices) and as necessary, the size of the bead can be deconvolved from the bead data, as DBT resolution might improve.^5^ The illustration in Fig. 6 shows a side view of a reversing (folded) bead ramp set which rise 10 mm in the z direction. The ramps on the right side of the phantom run opposite to the ramps on the left side of the phantom.

**Figure 7:**
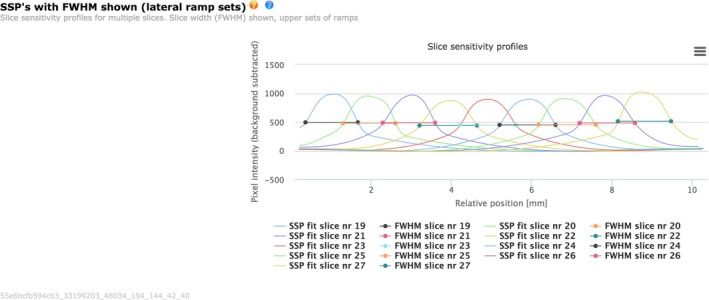
Slice Sensitivity Profiles (SSP's) FWHM as one moves through various slices (19–28).

The bead ramps are used to sample the Slice Sensitivity Profile (SSP) in the z domain, and result in the ability to plot single or multiple SSP's. Each SSP is sampled in‐plane by extracting the max intensity over the rows containing the bead ramp. A spline curve fitting is then applied to the peak locations from the profiles. Examples are shown in Fig. 7. The FWHM is determined from each of the SSP's of the numerical data. Good visual agreement is shown among 9 nominally contiguous slices.^9^

**Figure 8:**
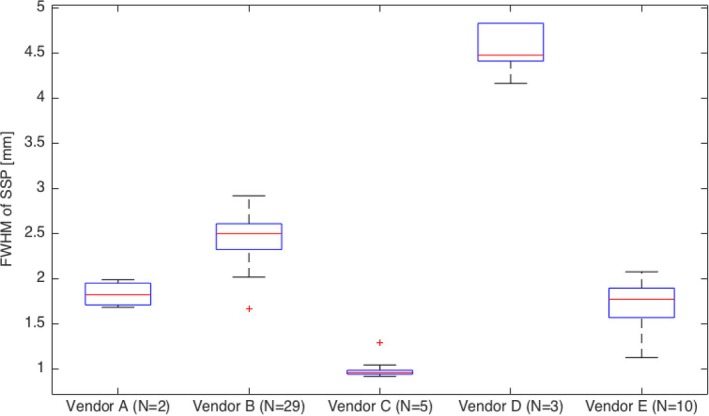
Data from five different vendors (a through e). Slice thickness range from 1 to 5 mm, depending on the vendor and the choice of acquisition parameters.

The phantom was used to test typical slice thickness as advocated by each of the five tested vendors A–E, with preliminary data shown in Fig. 8 for slices ranging from about 1 to 5 mm. The data were typically within 0.5 mm or better compared to the nominal slice width from each vendor. On each box, the central mark is the median, the edges of the box are the 25th and 75th percentiles, the whiskers extend to the most extreme data points the algorithm considers to be not outliers, and the outliers are plotted individually (red ‘+’ marker).

### 3.C Beads for z axis geometry

A related approach to studying z axis geometry is to study the distances and profiles of beads of known size and known vertical (z axis) spacing.^4^

**Figure 9:**
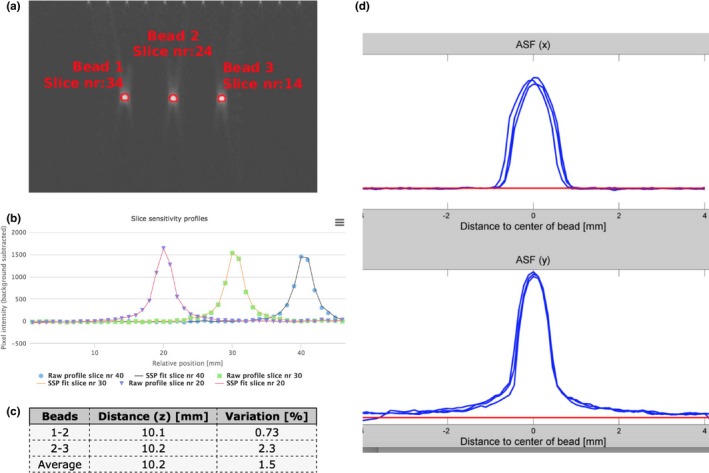
Beads for z‐axis geometry. Beads are offset by 10 mm in the z direction. (a) Image of three beads. (b) Slice Sensitivity Profiles (SSP's) for slice number 20, number 30, and number 40. (c) Calculated distance between the three beads, along with computed variation. (d) Resulting Artifact Spread Functions (ASF). Top is ASF (x) and bottom is ASF (y).

In the current phantom, three 5 mm Aluminum beads are nominally spaced 10 mm apart. The results from superimposing 3 different images, using these 5 mm bead sphere test objects are shown in Fig. 9: (a) images; (b) profile; (c) resulting spacing data.

### 3.D Artifact spread function

The same beads as used in the section Beads for z Axis Geometry can be used for what has been identified as the Artifact Spread Function (ASF)^2^, ^4^ is closely related to the SSP in the z axis direction. The ASF has been determined by calculating the z axis response of a small steel bead. In fact, the ASF was correlated with the convolution of the two‐dimensional (2D) point spread function (PSF) of the DBT system and the object function of the bead.^2^

In essence, the SSP from examining the sensitivity of adjoining small beads in the bead ramp supplies a function mimicking the results from the ASF.^4^ By examining Figs. 9(a)–9(c), it can be seen that as the z axis location of the bead changes, the ASF is generated. It can be noted that the SSP and ASF can vary not only depending on the x and y [Figs. 9(a) and 9(d)] but also vary depending on the z axis location of the bead as seen in Fig. 7.

### 3.E Spatial resolution

The same bead ramps provide a series of point sources (beads)^5^, ^9^, ^10^ These beads with their small diameter (18 mm) can be considered small enough to constitute “points” sources to determine the point spread function (PSF) and resulting MTF^2^, ^5^ for current levels of DBT resolution (typically 100 microns or better in DBT mode), particularly when one deconvolves the effective size of the small bead.^11^ It can be noted, that strictly speaking, the term MTF should be approached with caution for DBT, because the formal conditions for MTF are not met in systems that may be nonlinear and non‐Isoplanatic.^5^ Additionally, when one encounters iterative reconstructions in both Computed Tomography (CT), and DBT, the questions of linearity is even further strained and should probably be avoided. This being noted, the term has already been used in several studies 2, 10 and will be used in this paper, duly noting these caveats. The “MTF” data shown in this paper is obtained from the Fourier Transform of the PSF.^5^

**Figure 10:**
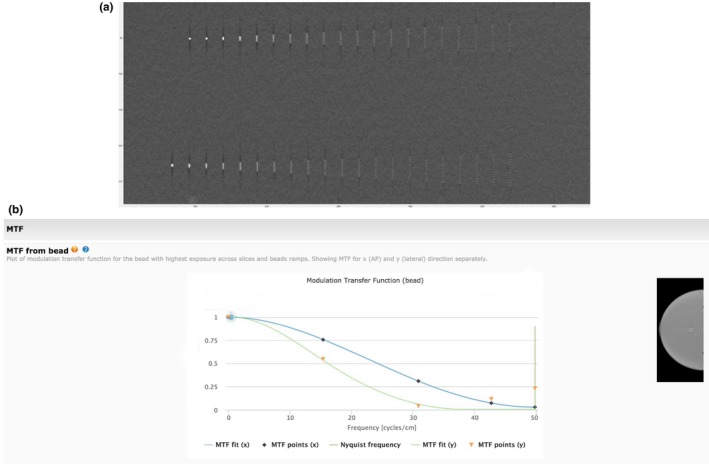
Use of small bead sources for point spread function and MTF. (a) Image of small beads in bead ramp used as Point Sources. Note the spreading of each bead increases as one moves from the center of the slice. (b) Corresponding MTF data. MTF (x) MTF (y) are shown.

A typical image for the beads (point sources) Fig. 10(a), on the bead ramps (Fig. 6); and the resulting MTF^10^ in both the x and y frequency directions is shown in Fig. 10(b). The resulting MTF plots show the known anisotropy between the MTF (x) and MTF (y) results due to the influence of the tube travel direction lowering the MTF in that direction.^12^ Anisotropic distributions can often lead to an MTF rising above the MTF (0) value^2^ Additional aspects of reconstruction filters and differences between In‐Plane MTF and other regions should be kept in mind http://www.aapm.org/meetings/amos2/pdf/42-11930-81688-724.pdf.

### 3.F Uniformity

A phantom of the same size and background composition as the DBT Phantom is available for checking the uniformity of the DBT response; however, it was decided to investigate the effectiveness of using the multi‐purpose phantom for DBT uniformity measurements instead of the dedicated uniformity phantom. The question is whether uniformity can be reasonably measured with the presence of the other test objects.^13^

**Figure 11:**
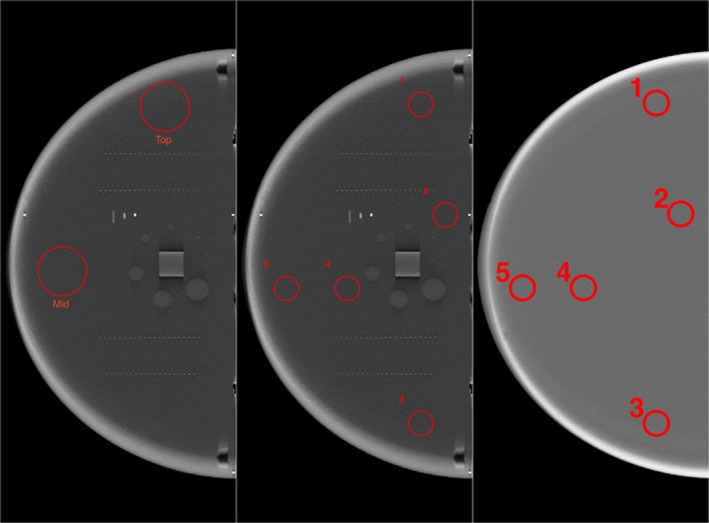
Images of Uniformity Section along with designated ROI's for Regional (two large areas), and global uniformity (five smaller ROI's).

Using the phantom both the regional uniformity and the global uniformity were studied. Uniform ROIs were carefully selected to minimize the effects of other test targets. For this study, we measure regional uniformity using two large (10 mm radius) regions of interest (ROIs), which are placed in anterior and lateral positions within each phantom image Fig. 11. To measure what we define as global uniformity, five ROIs, all with a radius of 5 mm are placed at various locations within slice. The means and standard deviations of the pixel values are computed. The max absolute difference between ROI means is a measure what we define as global uniformity.

**Figure 12:**
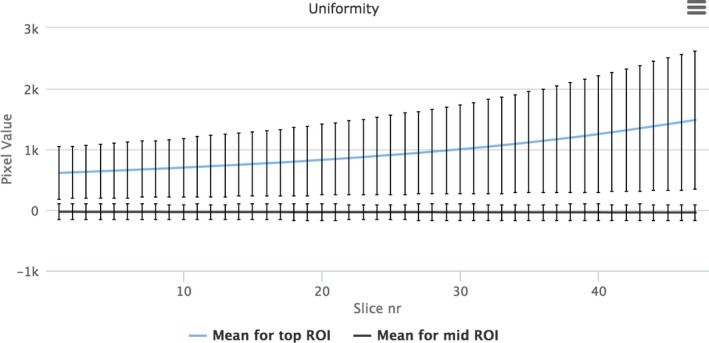
Uniformity variation for top ROI and mid ROI.

The two types of uniformity measurements were automatically computed for 49 scans from five different vendors. For regional uniformity, both the mean value and standard deviation of the pixel values within those regions were computed for all slices (z direction) see Fig. 12.

**Figure 13:**
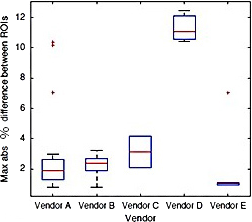
Global uniformity showing Vendor (A through E) comparison.

The global uniformity was computed on a dataset including a uniform phantom and the regular phantom. The global uniformity Fig. 13 showing vendor (A through E) comparison, a percentage difference was used as the pixel value scale is not normalized for Tomosynthesis systems. It is noted that unlike CT, where the Hounsfield scale provides a reasonably linear and portable relationship between x‐ray attenuation values and the output Hounsfield Units (HU), there is not yet an adopted scale of DBT units to other physical variables.^11^, ^13^

It can be noted that the uniformity measurements as defined in this paper, reveal differences between vendors, as well as trends across the slice dimension. However, these measurements in the multi‐purpose Tomophan QA phantom were limited to five 5 mm radius regions. A more detailed uniformity measurement can potentially be carried out using a solid uniformity phantom available as an option to the multipurpose phantom. This optional uniformity phantom is difference than the standard 14 mm tissue spacer that is included in the phantom (as seen in Fig. 2). No significant differences in global uniformity measurements were observed between the multipurpose Tomophan and the uniformity phantom. Therefore, by carefully selecting regions of interest, valuable information can be gained on Tomosynthesis image uniformity using a multipurpose QA phantom. These measurements were consistent with the values obtained with the optional solid uniformity phantom as previously presented. These findings on uniformity can potentially lead to significant time savings in administering a regular QA program whereby the multipurpose phantom can be used without necessarily using the optional solid uniform phantom. http://www.aapm.org/meetings/2015am/PRAbs.asp?mid=99&aid=28068.^13^

### 3.G Signal to noise ratio and contrast to noise ratio

**Figure 14:**
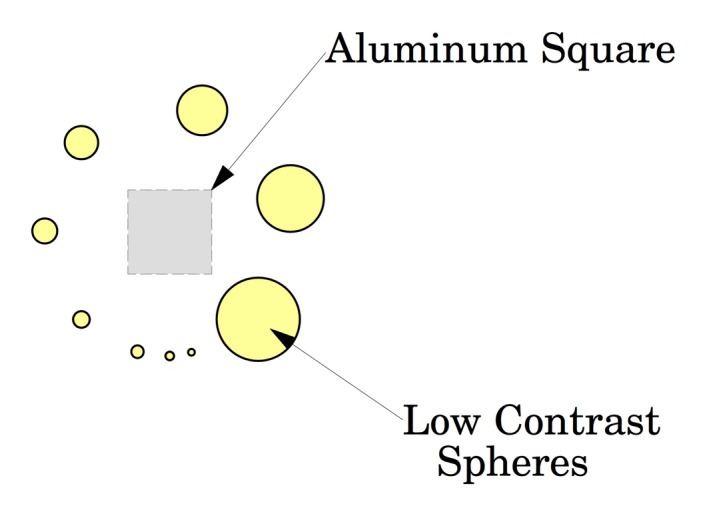
Low contrast tests spheres and Contrast to Noise Ratio (CNR) as measured over Aluminum square.

The Signal to Noise Ratio (SNR) can be obtained from taking the net signal over a uniform region and dividing by the standard deviation of the noise^6^ in a neighboring region; likewise, the CNR can be obtained by taking net signal over an Aluminum square and dividing by the standard deviation of the noise.^6^ The Aluminum square is found near the center of the phantom (just inside the circle of low contrast spheres) and is illustrated in Fig. 14 (schematic on left and scan on right).

**Figure 15:**
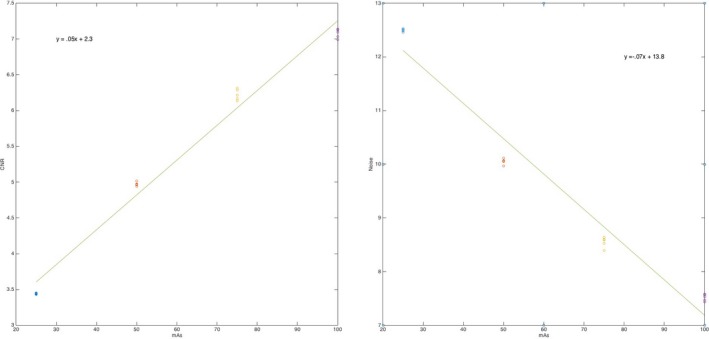
CNR and Noise data. CNR is shown on the left, and noise standard deviation on the right, both as a function of mAs.

A typical result (Fig. 15) from one vendor shows the decrease in noise (Standard Deviation)^6^ as the mAs is increased and the corresponding increase in CNR. In both cases, the fit equations show the approximate square root dependence of noise on mAs, and the corresponding effect on noise (SD) and CNR.^6^

### 3.H Low contrast

It can be noted that over and above the standard deviation of the noise, other higher order properties of the noise, such as Noise Power Spectrum (NPS) can be calculated.^6^ Thus, the assessment of the actual low contrast performance, can be subjective and controlled by the many caveats concerning the detectability of a given signal and parameters used to obtain the image.^11^ To extend the usefulness of the phantom to a test of visual detectability, a series of objects (spheres) are embedded in the phantom and are chosen to have a size and contrast which can be challenging to detectability in DBT. In the DBT Phantom, the low contrast objects are spheres with diameters of 0.8, 1, 1.5, 2, 3, 4, 6, 8, and 10 mm.

**Figure 16:**
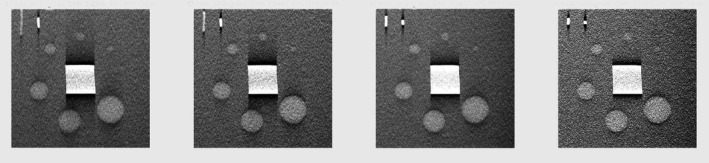
Examples of changes in acquisition parameters on the visualization of these spheres as shown in the four 37mAs images of figure 16; ranging from left of: 60°, CNR of 2.5; 48°, CNR 1.5; 24°, CNR 2.5; and 16°, CNR 1.1. One can also notice the decreased slice thickness with decreased number of views.

Examples of changes in acquisition parameters on the visualization of these spheres as shown in the four 37mAs images of Fig. 16; ranging from left of: 60°, CNR of 2.5; 48°, CNR 1.5; 24°, CNR 2.5; and 16°, CNR 1.1. One can also notice the decreased slice thickness with decreased number of views. These images are part of an independent study.^14^

### 3.I Remote analysis via the cloud

**Figure 17:**
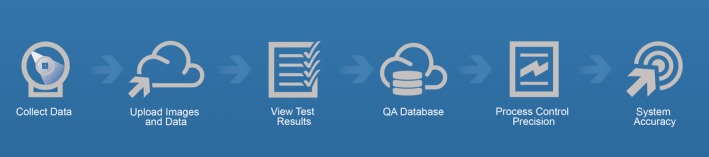
Overview of remote analysis via cloud with steps from left to right of: collection of data; uploading images and data; viewing test results; comparing with QA database; process control precision; system and phantom accuracy of alignment.

As discussed in this paper, all the data can be obtained from reports generated by remote analysis of DBT data from the Tomophan transmitted to analysis software via the cloud. In this paper, the data was provided by Image Owl https://www.imageowl.com/. The analysis can be seen in overview from Fig. 17 and involves the following steps: (a) collection of data, uploading images and data, viewing test results, comparing with QA database, process control precision, system and phantom accuracy of alignment. Examples of report can be obtained from Image Owl.

### 3.J Future work

**Figure 18:**
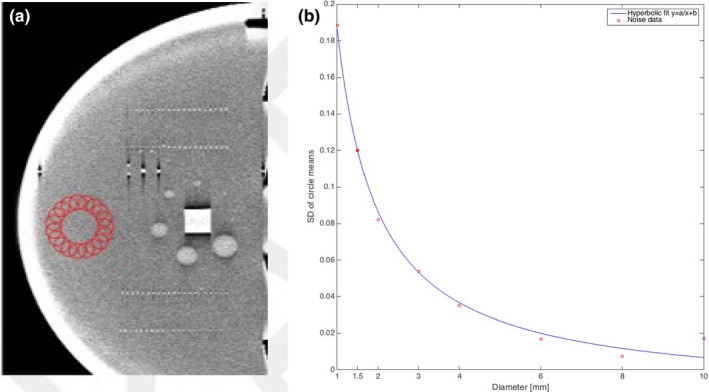
Illustration of method of noise calculations for bead sized regions. (a) Illustration of how noise is calculated in circles matching a given sphere diameter. Data collection scheme from one sphere diameter is shown in the figure. (b) Information showing noise data as a function of circle diameter where a hyperbolic fit is made to the resulting noise data as obtained from the noise standard deviation measured over circles of diameter matching the bead diameters.

Some initial work has been performed on modeling low contrast performance based on Contrast (C) – Detail (D), C‐D curves.^13^ For example, one approach involves extracting net (mean signal minus background) signals from circles with diameters equivalent to the diameters of the low‐contrast spheres [Fig. 18(a)]; from the uniform region, next to the low‐contrast spheres. Multiple circle means are sampled for each diameter. An example of sampling the values for 6 mm circles (spheres) is shown in Fig. 18(a). This image is from a slice through the phantom centered in a plane with the low contrast spheres. For each diameter (10, 8, 6, 4, 3, 2, 1.5 and 1 mm), one can then compute the standard deviation (SD) of these means, as shown in Fig. 18(a). In theory, the lower the diameter of the circle (sphere), the higher the SD of the means of the circles (more noise, less precision when using fewer pixels). Reference to other C‐D studies, often shows that a hyperbolic model will often fit these points.^11^ This seems to hold in the initial results shown in Fig. 18(b) where a hyperbolic fit is made to the resulting noise data as obtained from the noise standard deviation measured over circles of diameter matching the bead diameters.

This area of investigation will be expanded in future work, with more sophisticated models of detection, and possibly ROC analysis.^11^

## 4 CONCLUSIONS

The paper shows the design and initial test results of a phantom designed for Digital Breast Tomosynthesis systems. Illustrative examples of the use of the phantom are shown from several commercial systems, although no direct comparison is intended. The results show that a phantom can be designed not only to test the physical performance parameters of DBT systems but also, the phantom is amenable to automated analysis. The Image Owl Tomophan QA service offers automated image processing in the cloud allowing multiple users to review the same results from anywhere. Further benefits of the cloud include extensive data analysis and comparisons across similar types of equipment.

## CONFLICT OF INTEREST

David Goodenough is a Consultant to The Phantom Laboratory, Salem, NY. Joshua Levy is President and Founder of The Phantom Laboratory, Salem, NY. Hildur Ólafsdóttir is a member of Image Owl, Greenwich NY and Reykjavik, Iceland. Ingvi Olafsson is a member of Image Owl, Greenwich NY and Reykjavik, Iceland.
